# Perineural Administration of Dexmedetomidine in Axillary Brachial Plexus Block Provides Safe and Comfortable Sedation: A Randomized Clinical Trial

**DOI:** 10.3389/fmed.2022.834778

**Published:** 2022-05-17

**Authors:** Rihards P. Rocans, Agnese Ozolina, Mareks Andruskevics, Patrick Narchi, Diana Ramane, Biruta Mamaja

**Affiliations:** ^1^Clinic of Anaesthesiology, Riga East Clinical University Hospital, Riga, Latvia; ^2^Department of Anaesthesiology and Intensive Care, Riga Stradiņš University, Riga, Latvia; ^3^Anesthesia Department, Centre Clinical, Charente, France

**Keywords:** dexmedetomidine, axillary plexus brachialis block, sedation, wrist surgery, patient satisfaction

## Abstract

Dexmedetomidine prolongs the duration of regional block while its systemic sedative effect when administered perineurally is unknown. We aimed to evaluate the systemic sedative effect of perineural dexmedetomidine in patients after axillary brachial plexus block (ABPB). This single-blinded prospective randomized control trial included 80 patients undergoing wrist surgery receiving ABPB. Patients were randomized into two groups – Control group (CG, *N* = 40) and dexmedetomidine group (DG, *N* = 40). Both groups received ABPB with 20 ml of 0.5% Bupivacaine and 10 ml of 2% Lidocaine. Additionally, patients in DG received 100 mcg of dexmedetomidine perineurally. Depth of sedation was evaluated using Narcontrend Index (NI) and Ramsay Sedation Scale (RSS) immediately after ABPB and in several time points up to 120 min. Duration of block as well as patient satisfaction with sedation was evaluated using a postoperative survey. Our results showed that NI and RSS statistically differed between groups, presenting a deeper level of sedation during the first 90 min in DG compared to controls, *P* < 0.001. In the first 10 to 60 min after ABPB the median RSS was 4 (IQR within median) and median NI was 60 (IQR 44–80) in DG group, in contrast to CG patients where median RSS was 2 (IQR within median) and median NI was 97 (IQR 96–98) throughout surgery. The level of sedation became equal in both groups 90 and 120 min after ABPB when the median NI value was 98 (97–99) in DG and 97.5 (97–98) in CG, *P* = 0.276, and the median RSS was 2 (IQR within median) in both groups, *P* = 0.128. No significant intergroup differences in hemodynamic or respiratory parameters were found. Patients in DG expressed satisfaction with sedation and 86.5% noted that the sensation was similar to ordinary sleep. In DG mean duration of motor block was 13.5 ± 2.1 h and sensory block was 12.7 ± 2.8 h which was significantly longer compared to CG 6.3 ± 1.5 h, *P* < 0.001 and 6.4 ± 1.8 h, *P* < 0.001. We found that beside prolongation of analgesia, perineural administration of dexmedetomidine might provide rather safe and comfortable sedation with no significant effect on hemodynamic or respiratory stability and yields a high level of patient satisfaction.

## Introduction

The use of peripheral nerve blocks (PNB) has seen widespread adoption with the recent advancements of ultrasound-controlled techniques (1, 2). PNB provide adequate anesthesia for surgery, provide postoperative analgesia and decrease opioid requirements (3–7). Axillary brachial plexus block (ABPB) is the preferable option of anesthesia for wrist and hand surgery since it avoids the side effects of general anesthesia (8, 9).

Sedation is commonly applied in regional anesthesia. It is particularly useful for those who experience anxiety or restlessness and would prefer not to be awake during surgery (10). In order to choose the appropriate sedative agent, its side effects on spontaneous breathing and cardiovascular stability must be considered. Although midazolam is traditionally the most used sedative agent during regional anesthesia, alternative sedatives are emerging. dexmedetomidine is a highly selective α2 blocker which has recently gained widespread popularity due to its mild to moderate sedative, anxiolytic, and analgesic properties (11). During the last few years, increasing attention has been paid to reports demonstrating dexmedetomidine as a safe and effective sedative agent for intensive care (ICU) patients.

When dexmedetomidine is administered perineurally alongside local anesthetics it increases the duration of motor and sensory block (12–15). Curiously, previous reports have noted a systemic sedative effect after the perineural administration of dexmedetomidine (13, 16, 17) which was initially classified as an adverse effect.

We hypothesized that the systemic sedative effect produced by perineural Dexmedetomidine might have clear advantages during surgery under regional anesthesia. However, there is very limited data in previous literature on the systemic sedative effect of perineural dexmedetomidine. Therefore, our aim was to assess the systemic sedative effects of perineural administration of dexmedetomidine in patients receiving axillary brachial plexus block.

## Materials and Methods

The study protocol and the informed consent form were approved by the Ethics Committee of Riga East Clinical University hospital (Approval Number ZD/08-06/01-21/4). Written informed consent was obtained from every patient.

### Patient Selection and Patient Groups

Between 1^st^ of January and 31^st^ of May 2021, 86 consecutive adult patients were included in this single-blinded prospective randomized controlled study. All patients were admitted to the Latvian Microsurgery Center at Riga East University hospital, Riga, Latvia, to undergo urgent or elective wrist surgery.

The inclusion criteria: 18 years of age or older; ASA score of I–II. The exclusion criteria: pregnancy; history of mental or sleep disorders; sinus bradycardia (<50/min) just before performing ABPB; failed regional block (inadequate block 30 min after the attempt) and conversion to general anesthesia.

There were five patients excluded due to conversion to general anesthesia and one patient due to unexpected adverse effects related to the local anesthetics.

Simple randomization was performed by the researchers to allocate patients into two groups: control group (CG, *N* = 40) and dexmedetomidine group (DG, *N* = 40). The patients were included either in CG or DG group in a single-blinded manner.

### Perioperative Management

All patients received a premedication of 7.5 mg of oral midazolam (Dormicum®, F. Hoffman-La Roche AG, Switzerland) 30 min before transfer to the operating room. All patients underwent regional anesthesia with ABPB. The block was performed with the concurrent use of ultrasound and nerve stimulation guidance. The block was provided using 20 ml of 0.5% bupivacaine (Bupivacaine-Grindeks, AS Grindeks, Latvia) and 10 ml of 2% lidocaine (Lidocaine-Grindeks, AS Grindeks, Latvia) perineurally for patients in both groups. Additionally, patients in DG received 100 mcg of dexmedetomidine (Dexdor®, Orion Corporation, Finland) in 1 ml of normal saline perineurally. Standard monitoring with non-invasive blood pressure, pulse oximetry and heart rate was applied during surgery. The entire process of ABPB administration and intraoperative monitoring was carried out by a designated group of three experienced anaesthesiologists. Depth of sedation was continuously monitored using Narcotrend (Narcotrend Compact M, MT MonitorTechnik GmbH & Co. KG, Germany) which displays a derived electroencephalographic parameter referred to as the Narcotrend Index (NI). The Narcotrend Index is measured from 0 to 100 with values below 79 considered as light to moderate sedation and values below 64 considered as deep sedation or level of general anesthesia (18). Ramsay sedation scale (RSS) was also used to evaluate depth of sedation. RSS scores were assigned in the following manner: 1 point—patient is agitated; 2 points—patient is oriented and tranquil; 3 points—patient is arousable to verbal command; 4 points—patient is arousable to mild sensory stimulus; 5 points—patient has an incomplete reaction to painful stimulus; 6 points—patient has no reaction to painful stimulus. Values of Narcotrend Index (NI) and RSS score were obtained immediately after block, 10, 20, 30, 60, 90 and 120 min after the block as well as at the end of surgery.

The following conditions were defined as adverse effects: hypertension (systolic blood pressure >180 mmHg); tachycardia (heart rate >100/min at least 5 min); hypotension (mean arterial pressure <60 mmHg); bradycardia (heart rate <50/min at least 5 min); low oxygen saturation (SpO_2_ <90%). During surgery, patients with bradycardia (<50/min) received 0.5 mg of Atropine (Atropine Sopharma, Sopharma AD, Bulgaria) intravenously. Patients with low oxygen saturation (SpO_2_ <90%) were stabilized by securing the airway with head positioning and received oxygen *via* a nasal cannula or oxygen mask. Low oxygen saturation (SpO_2_ <90%) was the only designated indication for initiation of oxygen support.

A written postoperative survey was conducted on the first day after surgery after full recovery from sedation. The survey contained questions regarding the satisfaction with sedation and its similarity to ordinary sleep, the presence of postoperative nausea and the duration of sensory and motor block.

### Statistical Analysis

Statistical analysis was performed using the SPSS 26.0 (*Statistical Package for Social Sciences*). The Kolmogorov–Smirnov test was used to evaluate whether datasets conformed to normal distribution. Continuous variables were presented as mean ± standard deviation (SD) and categorical variables were presented as median ± IQR. Differences in data distribution between the groups were evaluated using Mann–Whitney *U* test for non-parametric datasets, and two-sample *t*-test or ANOVA for datasets conforming with normal distribution. Chi-square test was used for sets of nominal variables. Statistical significance was assumed if two-tailed *P* < 0.05.

## Results

### Clinical Course

In total 80 consecutive patients consisting of 38 men and 42 women were included. The mean age was 48.5 ± 14.9 years. All patients included in the study were scheduled for urgent or elective wrist surgery. There were no differences in age, gender distribution or ASA score, or body mass index between the groups, as depicted in Table 1. There were no significant intergroup differences in mean duration of block procedure either. Although, patients in the DG group had a shorter mean time to incision, the median time from end of block procedure to end of surgery was 120 min in both groups, with no significant intergroup difference, *P* = 0.096. As shown in Table 1, the CG had a significantly lower mean duration of postoperative sensory and motor block when compared to DG, *P* < 0.001.

**Table 1:** Demographic and clinical course characteristics of patients scheduled for wrist surgery undergoing axillary brachial plexus block.

	**Dexmedetomidine** **group *N* = 40**	**Control group** ***N* = 40**	***P*-Value**
**Age, years**	48.9 ± 17.3	48.0 ± 12.6	0.654
**Sex, female**, ***n*** **(%)**	22 (55)	20 (50)	0.779
**Body mass index**	24.1 ± 4.0	25.7 ± 6.3	0.159
**ASA score:**			
I class, *n* (%)	18 (45)	10 (25)	0.061
II class, *n* (%)	22 (55)	30 (75)	
**Wrist surgery type**			
Urgent, *n* (%)	8 (20)	10 (25)	0.592
Elective, *n* (%)	32 (80)	30 (75)	
**Duration of block procedure (min)**	10.0 ± 3.3	10.2 ± 3.1	0.764
**Time to incision (min)**	16.3 ± 3.4	20.8 ± 3.1	<0.001
**Duration of block (h)**			
Motory block	13.5 ± 2.1	6.4 ± 1.8	<0.001
Sensory block	12.7 ± 2.8	6.3 ± 1.5	<0.001

*Data are presented as mean ± SD or number (n) and percentage (%) and median (interquartile range).*

*SD, Standard Deviation; ASA, American Society of Anesthesiologists*.

Dominantly, patients were scheduled for elective wrist surgery. However, few urgent surgical cases were conformed to the study inclusion criteria. There was no significant difference in the proportion of elective and urgent patients between both groups, *P* = 0.790.

### Variables of Systemic Sedation Effect

**Figure 1:**
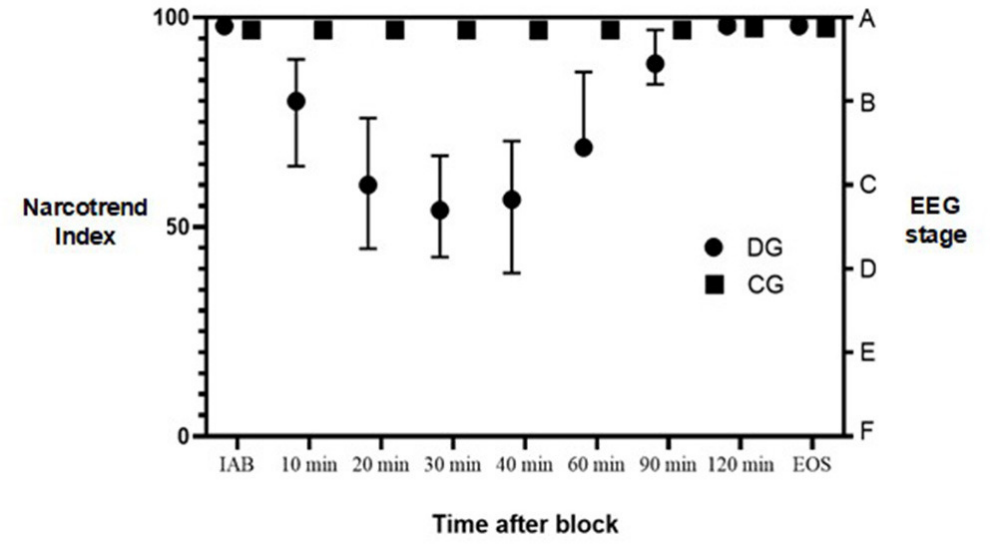
Narcotrend Index during surgery. DG, dexmedetomidine group; CG, control group; IAB, initially after block; EOS, end of surgery; EEG, electroencephalography. Dots represent median values. Lines represent interquartile range.

As shown in Figure 1, median values of NI were significantly lower in 10, 20, 30, 40 and 60 min after the ABPB in DG compared to controls, *P* < 0.001. Patients receiving dexmedetomidine perineurally demonstrated a median NI 98 (IQR, 97–99) immediately after the block. In 10 min, the median NI decreased to 80 (64.5–90), representing mild sedation. In the next 20 to 60 min median NI further decreased to a median of 57 (44–76), representing moderate to deep sedation. In 90 min, the median NI increased to 89 (84–97) when patients were mostly awake or mildly sedated. In contrast, patients in the CG remained wakeful and had a median NI of 97 (IQR 96–98) all throughout surgery. There ceased to be any statistically significant intergroup differences in NI values after 90 and 120 min.

Concomitantly, the intergroup statistical difference in median RSS score was found in 20–60 min after the ABPB, *P* < 0.001. Patients in the DG demonstrated RSS score 2 initially after block and then it increased to 4 in 20–60 min after block with the patient being sedated but easily awoken with verbal stimulus. Finally, in 90 min the median RSS score returned to 2. In contrast, patients in CG had a median RSS score of 2 initially after block and throughout surgery. There was no statistical difference in RSS score between the groups after 90 and 120 min.

### Variables of Respiratory and Hemodynamic Stability

**Figure 2:**
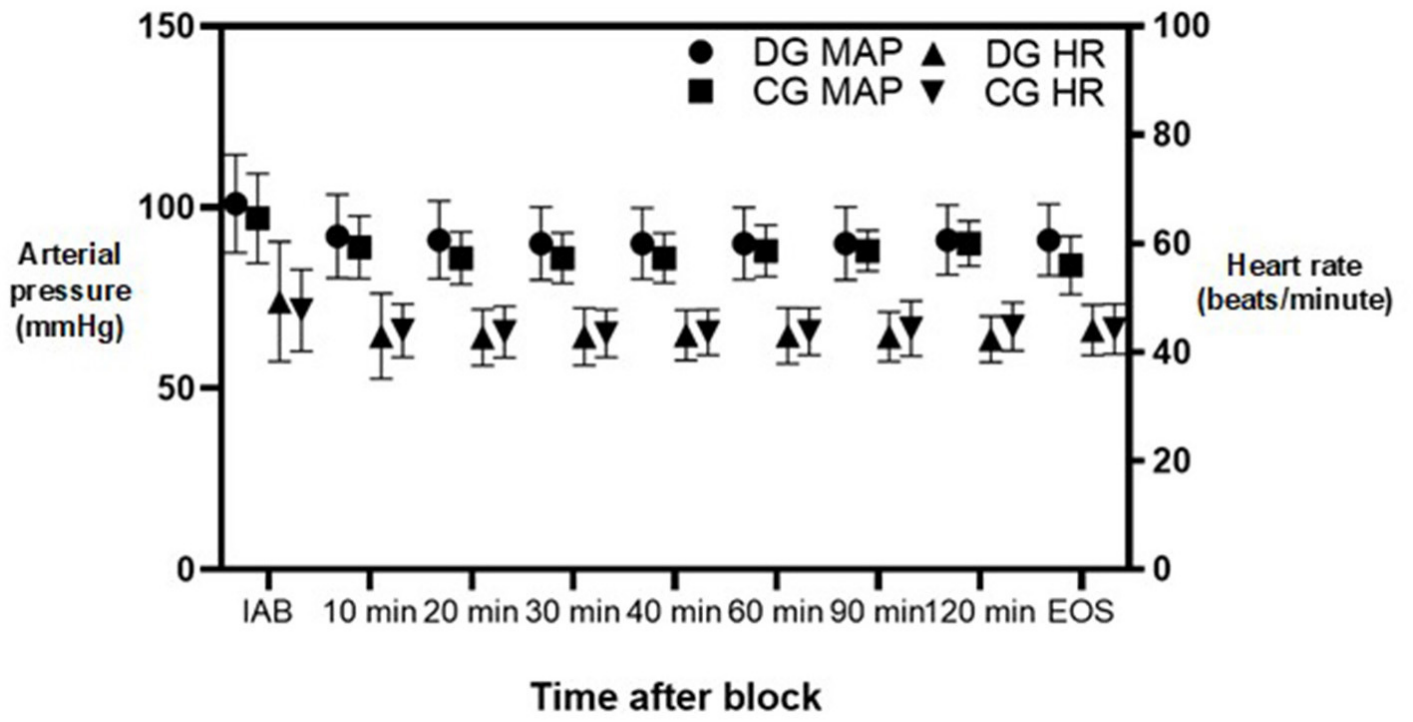
Mean arterial pressure and heart rate changes during surgery. DG, dexmedetomidine group; CG, control group; MAP, mean arterial pressure; HR, heart rate; IAB, initially after block; EOS, end of surgery. Dots represent median values. Lines represent standard deviation.

We found no differences in mean heart rate or mean arterial blood pressure (MAP) between the two groups all throughout the surgery. However, brief episodes of bradycardia were observed in 4 (10%) subjects from DG and 2 (5%) subjects from CG, *P* = 0.396. Changes in mean heart rate and MAP throughout surgery can be appreciated in detail in Figure 2.

Desaturation was rare in both groups. There was no statistical difference in median oxygen saturation all throughout surgery. However, a larger subset of subjects in the DG needed oxygen support by face mask to maintain adequate oxygenation (40% vs. 12.5%; *P* = 0.005).

### Patient Satisfaction

The survey revealed that 92.5% of subjects in the DG described falling asleep during surgery. Only 12.5% of patients in the CG recall sleeping during surgery. All patients in the DG expressed satisfaction with sedation and 86.5% of subjects found it comparable to ordinary sleep.

## Discussion

Dexmedetomidine has been recently proven to be an effective adjuvant to regional anaesthesia (12–15) Perineural administration of dexmedetomidine alongside local anesthetics is advantageous for prolonged surgery and provides long-duration postoperative analgesia (12, 17, 19). In the present study, DG patients demonstrated safe and comfortable systemic sedative effect after 100 mcg of dexmedetomidine added perineurally in ABPB for wrist surgery. Our most compelling finding was that NI and RSS statistically differed between both groups, suggesting a deeper level of systemic sedation during surgery in the first 90 min in DG patients compared to controls. Moreover, we noticed no significant events of hemodynamic instability in DG, confirming safe systemic sedation of dexmedetomidine when being administered perineurally. But so far, we suggest the proper monitoring must be applied since a larger subset of subjects in the DG needed oxygen support by face mask, especially if sicker patient is treated.

Dexmedetomidine was initially approved by the European Medicines Agency for use as a sedative in ICU setting (EMEA/H/C/002268). It has an acceptable tolerability profile, and its sedative effect is noninferior to other commonly used sedative agents in the ICU (20). At the same time, the administration of dexmedetomidine as an adjuvant to PNB is still considered as an off-label indication. This implies that both the local and systemic effects of perineural administration are yet to be fully examined.

Nevertheless, multiple authors have previously proposed adding dexmedetomidine as an adjuvant to local anesthetics for prolongation of sensory and motor blockade (14, 17). A systematic review by El-Boghdadly and co-authors found perineural dexmedetomidine to be a more effective adjuvant than Clonidine (14). In contrast, Albrecht and co-authors found that perineural dexmedetomidine was a less effective adjuvant than dexamethasone (21). The previously stated publications on perineural dexmedetomidine have noted the appearance of side effects such as systemic sedation and bradycardia (14, 16, 17, 21). The intravenous administration of dexmedetomidine has also been proven to be equally effective as compared to perineural dexmedetomidine with respect to onset and duration of block and duration of analgesia but has greater hemodynamic instability (22). We attempted to demonstrate that the systemic sedative effect of perineural dexmedetomidine can be objectively measured and is in fact beneficial in the context of regional anesthesia. Furthermore, we attempted to demonstrate that patient safety and satisfaction might be achieved with the appropriate dosing strategy.

Several studies have focused on multiple dosing strategies. A meta-analysis of 32 studies by Vorobeichik and co-authors suggest 50–60 mcg of perineural dexmedetomidine to be the optimal dose for prolongation of sensory blockade while avoiding hemodynamic instability (17). A meta-analysis of 12 studies by Dai and co-authors did not find a significant difference in incidence of hemodynamic or respiratory instability between doses <50 mcg and >50 mcg (23). It has been found that a dexmedetomidine plasma concentration of 0.2–0.3 ng/ml provides moderate systemic sedation (11). A prospective study by Fritsch and co-authors revealed that 150 mcg of perineural dexmedetomidine led to a plasma concentration of 0.37 ng/ml in 90 min (12) which exceeds the previously stated plasma concentration for moderate sedation. So far, a study by Keplinger and co-authors has concluded that the 100 mcg dose level for perineural dexmedetomidine may represent an optimal balance between efficacy and sedation (16). Based on previous reports, we considered that 100 mcg of perineural dexmedetomidine might provide safe systemic sedation during surgery and would not have any marked effects on hemodynamic or respiratory stability.

Two randomized groups were included in our study and proved to be indistinguishable by population characteristics, ASA score and surgical factors and thus further comparisons were not at risk of confounding factors.

In our study the Narcotrend Index (NI) was used as objective criteria to assess the depth of sedation. As far as we know, there is only one study that uses NI to explore the sedative effect of dexmedetomidine in the context of continuous epidural anesthesia (24). There are no previous studies which have used NI to measure the systemic sedative effect of perineural dexmedetomidine in PNB.

When assessing patient satisfaction, we found that every patient in the DG expressed satisfaction with sedation and most of patients compared the systemic sedative effect to ordinary sleep. Clinicians often report that it would be preferable if during the sedation the patient could be easily awoken with verbal stimulus and be oriented and cooperative. Such effects of dexmedetomidine have already been elucidated in previous studies (19, 25). This implies that perineural dexmedetomidine has a high potential for patient and clinician satisfaction. It must be noted that in our study most subjects spent a considerable amount of surgical time with no sedation since the duration of sedation provided by perineural Dexmedetomidine using this dosing strategy was only 90 min. Admittedly, the surgery ended in less than 90 min after the PNB in only 20% of cases. Furthermore, 20–60 min after ABPB the NI and RSS indicated moderate to deep sedation which may exceed the necessary depth of sedation for surgery under regional anesthesia. Therefore, it is too early to conclude that 100 mcg is the optimal dose for effective and safe systemic sedation since in some cases the time from block until end of surgery exceeds 90 min.

Previous data on the systemic complications of perineural dexmedetomidine are similar to those observed with its intravenous administration, with the main complications being hypotension and bradycardia. No serious adverse effects of 100 mcg perineural dexmedetomidine were noted in our study. As mentioned before, a larger subset of subjects in the DG received oxygen support *via* face mask which may emphasize the need for diligent monitoring of SpO_2_ during the sedative effect, although no significant events of respiratory instability requiring airway establishment were otherwise noted. There was no statistical difference in median oxygen saturation, mean heart rate or MAP all throughout surgery. Our observations are consistent with recent findings of investigators, who reported that hemodynamic changes caused by perineural dexmedetomidine were not found to be dose-dependent and were not severe enough to warrant the use of hemodynamic support (23). Moreover, a systematic review by Barends et al. (25) showed that intravenous dexmedetomidine during procedural sedation has advantages over midazolam in terms of reliability, analgesia and patients' and clinicians' satisfaction while maintaining a similar cardio-respiratory safety profile as well.

Additionally, we found perineural dexmedetomidine to prolong the duration of sensory block by 6.4 h and motor block by 7.1 h. A meta-analysis by Vorobeichik and co-authors revealed a more substantial prolongation of duration of sensory block 7.7–11.5 h and motor block 6.9–10.1 h (17). Apart from the previously known fact that dexmedetomidine prolongs PNB, this discrepancy might be partially explained by the fact that the duration of sensory and motor block was evaluated by the postoperative survey instead of an objective assessment by the clinician.

### Limitations

Our study was not conducted in a double-blind manner, therefore, clinician awareness of Dexmedetomidine administration may have influenced some of our results. However, this might be slightly mitigated by the fact that the entire process of ABPB administration and intraoperative monitoring was carried out by a designated team of three experienced anaesthesiologists instead of just a single clinician. Moreover, NI as an objective criterion was used to assess the depth of sedation.

Another limitation is the fact that the duration of sensory and motor block was provided by the subject filling the postoperative survey instead of an objective assessment by the clinician. This might have affected the results, reporting shorter duration of sensory and motor block since the patient might have felt a subjective regain of function while there still might be objective signs of residual blockade.

Since it is our common practice to provide premedication with 7.5 mg of oral midazolam, we should take into consideration the fact, that all patients received premedication, also in DG. Thus, the subsequent sedative effect from perineural dexmedetomidine might be slightly affected by the residual effects of the premedication. We speculate that possible side effects, particulary on hemodynamic function, of perineural 100 mcg dexmedetomidine could be more harmful in patients with pre-existing conditions or advanced age, since only ASA I and II patients were included in our study. Therefore, the dose and indication for dexmedetomidine sedation effect should be individually evaluated.

Despite these limitations, our results indicate that 100 mcg of perineural dexmedetomidine provides rather safe and effective sedation without significantly affecting respiratory or hemodynamic stability. Moreover, with this dose of dexmedetomidine, subjects had no additional requirement for intravenous sedation during surgery. Patients most commonly associate this type of sedation with the sensation of ordinary sleep and express a high level of satisfaction.

In conclusion, we found that perineural administration of 100 mcg of dexmedetomidine in axillary brachial plexus block might provide rather safe systemic sedation with no significant effect on hemodynamic or respiratory stability and yields a high level of patient satisfaction.

## Data Availability Statement

The raw data supporting the conclusions of this article will be made available by the authors, without undue reservation.

## Ethics Statement

The studies involving human participants were reviewed and approved by Ethics Committee of Riga East Clinical University hospital (Approval Number ZD/08-06/01-21/4). The patients/participants provided their written informed consent to participate in this study.

## Author Contributions

AO, RR, and MA designed and directed the trial. DR and MA performed the trial and collected data. RR performed statistical analysis. AO and RR developed the theoretical framework and wrote the manuscript in consultation with PN and BM. All authors contributed to the article and approved the submitted version.

## Funding

The authors declare that Riga Stradiņš University kindly covered the publication fee for this article. The funder was not involved in the study design, collection, analysis, interpretation of data or writing of this article.

## Conflict of Interest

The authors declare that the research was conducted in the absence of any commercial or financial relationships that could be construed as a potential conflict of interest.

## Publisher's Note

All claims expressed in this article are solely those of the authors and do not necessarily represent those of their affiliated organizations, or those of the publisher, the editors and the reviewers. Any product that may be evaluated in this article, or claim that may be made by its manufacturer, is not guaranteed or endorsed by the publisher.

## References

[1] Perlas A Brull R Chan VW McCartney CJ Nuica A Abbas S. Ultrasound guidance improves the success of sciatic nerve block at the popliteal fossa. Reg Anesth Pain Med. (2008) 33:259–65. 10.1016/j.rapm.2007.10.010 18433678

[2] Chan VW Perlas A McCartney CJ Brull R Xu D Abbas S. Ultrasound guidance improves success rate of axillary brachial plexus block. Can J Anaesth. (2007) 54:176–82. 10.1007/BF03022637 17331928

[3] Joshi G Gandhi K Shah N Gadsden J Corman SL. Peripheral nerve blocks in the management of postoperative pain: challenges and opportunities. J Clin Anesth. (2016) 35:524–9. 10.1016/j.jclinane.2016.08.041 27871587

[4] Memtsoudis SG Poeran J Cozowicz C Zubizarreta N Ozbek U Mazumdar M. The impact of peripheral nerve blocks on perioperative outcome in hip and knee arthroplasty-a population-based study. Pain. (2016) 157:2341–9. 10.1097/j.pain.0000000000000654 27643835

[5] Chan EY Fransen M Parker DA Assam PN Chua N. Femoral nerve blocks for acute postoperative pain after knee replacement surgery. Cochrane Database Syst Rev. (2014) 2014:CD009941. 10.1002/14651858.CD009941.pub2 24825360 PMC7173746

[6] Ullah H Samad K Khan FA. Continuous interscalene brachial plexus block versus parenteral analgesia for postoperative pain relief after major shoulder surgery. Cochrane Database Syst Rev. (2014) 2014:CD007080. 10.1002/14651858.CD007080.pub2 24492959 PMC7182311

[7] Xu J Chen XM Ma CK Wang XR. Peripheral nerve blocks for postoperative pain after major knee surgery. Cochrane Database Syst Rev. (2014) CD010937. 10.1002/14651858.CD010937 25501884

[8] O'Donnell BD Ryan H O'Sullivan O Iohom G. Ultrasound-guided axillary brachial plexus block with 20 milliliters local anesthetic mixture versus general anesthesia for upper limb trauma surgery: an observer-blinded, prospective, randomized, controlled trial. Anesth Analg. (2009) 109:279–83. 10.1213/ane.0b013e3181a3e721 19535722

[9] Chan VW Peng PW Kaszas Z Middleton WJ Muni R Anastakis DG. comparative study of general anesthesia, intravenous regional anesthesia, and axillary block for outpatient hand surgery: clinical outcome and cost analysis. Anesth Analg. (2001) 93:1181–4. 10.1097/00000539-200111000-00025 11682392

[10] Borgeat A Aguirre J. Sedation and regional anesthesia. Curr Opin Anaesthesiol. (2009) 22:678–82. 10.1097/ACO.0b013e32832f3320 19606025

[11] Weerink MAS Struys MMRF Hannivoort LN Barends CRM Absalom AR Colin P. Clinical pharmacokinetics and pharmacodynamics of dexmedetomidine. Clin Pharmacokinet. (2017) 56:893–913. 10.1007/s40262-017-0507-7 28105598 PMC5511603

[12] Fritsch G Danninger T Allerberger K Tsodikov A Felder TK Kapeller M. Dexmedetomidine added to ropivacaine extends the duration of interscalene brachial plexus blocks for elective shoulder surgery when compared with ropivacaine alone: a single-center, prospective, triple-blind, randomized controlled trial. Reg Anesth Pain Med. (2014) 39:37–47. 10.1097/AAP.0000000000000033 24317234

[13] Shaikh SI Mahesh SB. The efficacy and safety of epidural dexmedetomidine and clonidine with bupivacaine in patients undergoing lower limb orthopedic surgeries. J Anaesthesiol Clin Pharmacol. (2016) 32:203–9. 10.4103/0970-9185.182104 27275050 PMC4874075

[14] El-Boghdadly K Brull R Sehmbi H Abdallah FW. Perineural dexmedetomidine is more effective than clonidine when added to local anesthetic for supraclavicular brachial plexus block: a systematic review and meta-analysis. Anesth Analg. (2017) 124:2008–20. 10.1213/ANE.0000000000002014 28525514

[15] Andersen JH Jaeger P Grevstad U Estrup S Geisler A Vilhelmsen F. Systemic dexmedetomidine is not as efficient as perineural dexmedetomidine in prolonging an ulnar nerve block. Reg Anesth Pain Med. (2019) 44:333–40. 10.1136/rapm-2018-100089 30679332

[16] Keplinger M Marhofer P Kettner SC Marhofer D Kimberger O Zeitlinger M. Pharmacodynamic evaluation of dexmedetomidine as an additive drug to ropivacaine for peripheral nerve blockade: a randomised, triple-blind, controlled study in volunteers. Eur J Anaesthesiol. (2015) 32:790–6. 10.1097/EJA.0000000000000246 25695189

[17] Vorobeichik L Brull R Abdallah FW. Evidence basis for using perineural dexmedetomidine to enhance the quality of brachial plexus nerve blocks: a systematic review and meta-analysis of randomized controlled trials. Br J Anaesth. (2017) 118:167–81. 10.1093/bja/aew411 28100520

[18] Weber F Pohl F Hollnberger H Taeger K. Impact of the Narcotrend Index on propofol consumption and emergence times during total intravenous anaesthesia with propofol and remifentanil in children: a clinical utility study. Eur J Anaesthesiol. (2005) 22:741–7. 10.1017/S0265021505001237 16211731

[19] Li J Wang H Dong B Ma J Wu X. Adding dexmedetomidine to ropivacaine for femoral nerve block inhibits local inflammatory response. Minerva Anestesiol. (2017) 83:590–7. 10.23736/S0375-9393.17.11430-6 28106354

[20] Keating GM. Dexmedetomidine: a review of its use for sedation in the intensive care setting. Drugs. (2015) 75:1119–30. 10.1007/s40265-015-0419-5 26063213

[21] Albrecht E Vorobeichik L Jacot-Guillarmod A Fournier N Abdallah FW. Dexamethasone is superior to dexmedetomidine as a perineural adjunct for supraclavicular brachial plexus block: systematic review and indirect meta-analysis. Anesth Analg. (2019) 128:543–54. 10.1213/ANE.0000000000003860 30303864

[22] Somsunder RG Archana NB Shivkumar G Krishna K. Comparing efficacy of perineural dexmedetomidine with intravenous dexmedetomidine as adjuvant to levobupivacaine in supraclavicular brachial plexus block. Anesth Essays Res. (2019) 13:441–5. 10.4103/aer.AER_105_19 31602059 PMC6775837

[23] Dai W Tang M He K. The effect and safety of dexmedetomidine added to ropivacaine in brachial plexus block: a meta-analysis of randomized controlled trials. Medicine. (2018) 97:e12573. 10.1097/MD.0000000000012573 30313043 PMC6203584

[24] Li CX Li H. [Determination of the 50% effective concentration of dexmedetomidine as an adjuvant in combined spinal-epidural anesthesia with Narcotrend]. Nan Fang Yi Ke Da Xue Xue Bao. (2011) 31:734–6. 21515484

[25] Barends CR Absalom A van Minnen B Vissink A Visser A. Dexmedetomidine versus midazolam in procedural sedation. A systematic review of efficacy and safety. PLoS ONE. (2017) 12:e0169525. 10.1371/journal.pone.0169525 28107373 PMC5249234

